# Myxovirus Resistance Protein A mRNA Expression Kinetics in Multiple Sclerosis Patients Treated with IFNβ

**DOI:** 10.1371/journal.pone.0169957

**Published:** 2017-01-12

**Authors:** Jana Libertinova, Eva Meluzinova, Ales Tomek, Dana Horakova, Ivana Kovarova, Vaclav Matoska, Simona Kumstyrova, Miroslav Zajac, Eva Hyncicova, Petra Liskova, Eva Houzvickova, Lukas Martinkovic, Martin Bojar, Eva Havrdova, Petr Marusic

**Affiliations:** 1 Department of Neurology, Charles University, 2nd Faculty of Medicine and Motol University Hospital, Prague, Czech Republic; 2 Department of Neurology and Center of Clinical Neuroscience, Charles University, First Faculty of Medicine and General University Hospital, Prague, Czech Republic; 3 Laboratory of Molecular Diagnostics, Na Homolce Hospital, Prague, Czech Republic; 4 Department of Medical Microbiology, Charles University, 2nd Faculty of Medicine and Motol University Hospital, Prague, Czech Republic; San Raffaele Scientific Institue, ITALY

## Abstract

**Introduction:**

Interferon-β (IFNß) is the first-line treatment for relapsing-remitting multiple sclerosis. Myxovirus resistance protein A (MxA) is a marker of IFNß bioactivity, which may be reduced by neutralizing antibodies (NAbs) against IFNß. The aim of the study was to analyze the kinetics of MxA mRNA expression during long-term IFNβ treatment and assess its predictive value.

**Methods:**

A prospective, observational, open-label, non-randomized study was designed in multiple sclerosis patients starting IFNß treatment. MxA mRNA was assessed prior to initiation of IFNß therapy and every three months subsequently. NAbs were assessed every six months. Assessment of relapses was scheduled every three months during 24 months of follow up. The disease activity was correlated to the pretreatment baseline MxA mRNA value. In NAb negative patients, clinical status was correlated to MxA mRNA values.

**Results:**

119 patients were consecutively enrolled and 107 were included in the final analysis. There was no correlation of MxA mRNA expression levels between baseline and month three. Using survival analysis, none of the selected baseline MxA mRNA cut off points allowed prediction of time to first relapse on the treatment. In NAb negative patients, mean MxA mRNA levels did not significantly differ in patients irrespective of relapse status.

**Conclusion:**

Baseline MxA mRNA does not predict the response to IFNß treatment or the clinical status of the disease and the level of MxA mRNA does not correlate with disease activity in NAb negative patients.

## Introduction

Interferon-β (IFNβ) is one of the first-line treatments in patients with clinically isolated syndrome (CIS) or relapsing-remitting multiple sclerosis (RR MS). Nevertheless, not all patients respond well to long-term IFNβ therapy [1]. Patients who do not respond or who relapse can be classified as either: 1. pathogenesis related non-responders, when IFNβ treatment has a low ability to suppress the high disease activity, or 2. immunopharmacological non-responders, when IFNβ treatment fails due to the presence of neutralizing antibodies (NAbs) against IFNβ, preventing the interaction between IFNβ and its receptor, thus causing the loss of IFNβ bioactivity [2].

The biological activity of IFNβ can be estimated by measuring specific biomarkers known to be downstream of IFNβ signaling. Myxovirus resistance protein A (MxA) belongs to the group of IFNβ induced proteins and gene expression of this protein has proven to be one of the most reliable biomarkers of IFNβ bioactivity [2]. MxA mRNA level dramatically increases after the initiation of IFNβ treatment [3].

Recent data has confirmed the relationship between IFNβ bioactivity and clinical disease activity, showing that high MxA mRNA levels in treated patients are related to lower relapse rates and to clinical stabilization of the disease [4; 5; 6]. Conversely, a significant drop in MxA mRNA level during treatment indicates a decrease or even loss of IFNβ biological activity [7]. This loss of activity is mostly caused by the production of NAbs [2; 5]. However, there are some patients treated with IFNβ that manifest a decrease in MxA mRNA without NAb production—this suggests that there may be another group of non-responders with a novel mechanism underlying the decrease in IFNβ biological activity [8; 9; 5].

There is evidence to show that baseline levels of IFN stimulated genes can predict the response to IFNβ treatment [10; 11] or that baseline level is a marker of the clinical activity of the disease [12; 13].

The aims of our study were to; 1. analyze the kinetics of MxA mRNA levels during long-term IFNβ treatment in relation to the clinical course of the disease and to NAb production, and 2. assess the predictive value of baseline MxA mRNA level for the response to IFNβ treatment and the clinical course of the disease.

## Methods

### Study design

A prospective, observational, open-label, non-randomized study was conducted in collaboration with two Multiple Sclerosis Centres at the University Hospitals in Prague. The patient enrolment was started in June 2011 and completed in January 2013. Treatment naive patients presenting with CIS (suggestive of multiple sclerosis development), or diagnosed with RR MS, fulfilling the McDonald criteria [14], were included. After enrolment, the patients started treatment of either, IFNβ-1a intramuscularly at 30 μg once a week, IFNβ-1a subcutaneously at 22 or 44 μg three times per week or IFNβ-1b subcutaneously at eight million IU every other day. Patients were not randomized to the treatment; medication was selected according to the common medical practice in the Centres.

The duration of the follow-up was 24 months. Clinical assessment (relapse rate, Expanded Disability Status Scale—EDSS) was performed at baseline and every three months thereafter.

### Samples

Blood samples were obtained before the first IFNβ injection (at baseline) and every three months subsequently, 12±3 hours after the last IFNß injection and only when there was no evidence of concomitant infection.

MxA mRNA was assessed by real-time PCR in peripheral blood mononuclear cells. Blood samples were collected in EDTA tubes or TEMPUS Blood RNA tubes (ThermoFisher scientific) according to the manufacturer’s transport and storage recommendations (TEMPUS tubes allow a delay of ≥24 hours between sample collection and RNA isolation if storage and transport is at 4°C). RNA extraction from EDTA collection tubes was performed with the QIAmp RNA blood Mini Kit (Quiagen) according to the manufacturer’s protocol. Isolation from TEMPUS tubes was undertaken using Tempus Spin RNA isolation kits (Applied Biosytems/ ThermoFisher scientific). The RNA concentrations in isolates were measured by a DeNovix D11- Spectrophotometer. Subsequent cDNA transcription and qPCR was done using EXPRESS One-Step SYBR^®^ GreenER^™^ Universal Kits (Invitrogen/ Thermo Fisher Scientific) according to the manufacturer’s protocol. PCR primers have been published elsewhere [6]. Reactions were performed on Rotor-Gene (Corbett Research) and Rotor-Gene Q (Quiagen) in duplicate. We set up a comparative quantification assay [15] to assess the expression of MxA mRNA relative to GAPDH (glyceraldehyde-3-phosphate dehydrogenase) expression (according to the Rotor-Gene and Rotor-Gene Q manual "Comparative Quantification").

NAbs were determined every six months using the antiviral cytopathic effect assay [16]. A level of 20 TRU/ml was considered to be the threshold for NAb positivity [17].

In our study, patients with reduced bioactivity of IFNβ, i.e. NAb positive and/or with a MxA mRNA level below the cut off (confirmed in repeated tests), were labeled as IFNβ biological non-responders. Patients without these laboratory findings were considered to be biological responders.

Relapse was defined as a new neurological symptom or as a worsening of a previous one, accompanied by consistent neurological dysfunction lasting for at least 24 hours in an afebrile patient. Clinical activity was determined by ‘relapse rate’, i.e. the number of relapses over one year. For the survival analysis we used the ‘time to the first relapse’. Disability progression was defined as an increase in the EDSS by 1.0 point (if the baseline EDSS>0) or 1.5 point (if the baseline EDSS = 0), confirmed after 6 months. Patients were classified as clinical responders when there was no relapse and no EDSS progression during the whole follow up period.

The study protocol was approved by the Ethics Committee of Motol University Hospital and each participating patient gave written informed consent.

### Statistical analysis

Two cut off values of MxA gene expression were used in the study: 1) the pretreatment baseline value and 2) the treatment efficacy value.

The optimal cut off value for the pretreatment baseline MxA was determined by two techniques: ROC (Receiver Operating Characteristics) analysis with area under the curve (AUC) analysis and secondly using median, tercile and quartile of the baseline MxA mRNA values. All four respective values were used in the Cox proportional model as variables.

The cut off value reflecting an optimal reaction to IFNβ treatment (treatment efficacy value) was acquired during the period of method validation. The cut off value was calculated as the mean MxA value plus two standard deviations (95th percentile) of control samples (a set of 105 patients with RR MS or CIS, not treated with IFNβ). An MxA mRNA value lower than this cut off in IFNβ treated patients was interpreted as demonstrating inefficacy of the IFNβ treatment.

The Cox proportional hazards model was used for the risk of relapse (time to first relapse on the treatment) calculation. Correlations were performed using Pearson and Spearman rank correlation. Results were considered statistically significant if p < 0.05. All statistical analyses were performed using IBM SPSS Statistics 22 software (IBM Corporation, Armonk, New York, USA) and GraphPad Prism software 4.0 (GraphPad Software, San Diego, USA).

## Results

### The study population and their MxA and NAb profile

In total, 119 patients, 43 men, 76 women, median age at the onset 33 years (19–65 years), were consecutively enrolled. There were 54 patients diagnosed with CIS and 65 patients with RR MS at the beginning, mean baseline EDSS was 1.9 (0–4.5). In 38 patients, treatment with IFNβ-1a i.m. had been started, 42 patients had been treated with IFNβ-1a s.c. and 39 patients with IFNβ-1b s.c. During the course of the study, 12 patients were excluded; the reasons for termination are given in Table 1.

**Table 1:** Reasons for exclusion from the study.

Reason for exclusion	No. of patients	Duration of IFNβ treatment in individual patient (months)
Adverse effect of IFNβ	4	4; 7; 11; 17
Pregnancy	3	9; 12; 21
Non-compliance	5	3; 11; 12; 20; 23

Out of 107 remaining subjects, 14 patients were switched from IFNβ to an alternative treatment during the study; eight of them due to disease activity and six due to repeated NAb positivity. The detailed clinical and demographic characteristics of the analyzed cohort are given in Table 2.

**Table 2:** Clinical and demographic characteristics of the analysed cohort (n = 107).

Variable	Total (n = 107)	NAb negative (n = 90)	NAb positive (n = 17)	Clinical responders (n = 42)	Clinical non-responders (n = 65)
Female (%)	61.7%	61.1%	64.7%	55.4%	71.4%
Age (mean ± SD)*	34.6 **±** 9.1	34.2 **±** 8.5	36.7 **±** 11.5	35.5 **±** 9.7	33.3 **±** 7.8
EDSS (mean ± SD)	1.9 **±** 0.9	1.9 **±** 0.9	2.1**±** 0.9	1.9 **±** 0.8	2.1**±** 0.9
Disease duration** (mean ± SD)	44 **±** 71	43 **±** 64	47 **±** 102	46 **±** 77	42 **±** 62

*Age in years at the initiation of IFNβ treatment (study baseline)

** Disease duration in months from the first symptom to the initiation of IFNβ treatment (study baseline)

### Clinical course of the disease

54 patients classified as CIS were enrolled into the study. During the 24-month follow-up 51 (94%) progressed into clinically definitive RR MS. EDSS confirmed progression was documented in 11 patients, three were patients with a loss of IFNβ efficacy (MxA mRNA below the cut off) and the progression of the disability was related to the relapse in all. In the remaining eight patients the bioactivity of IFNβ was preserved and NAbs were negative.

### NAb and MxA mRNA expression kinetics

In all patients the expression of MxA mRNA levels significantly increased after initiation of IFNβ treatment, the difference between month zero (baseline) and month three was 610.7 on average (95% CI 544.8–676.7, p < 0.001).

**Fig 1:**
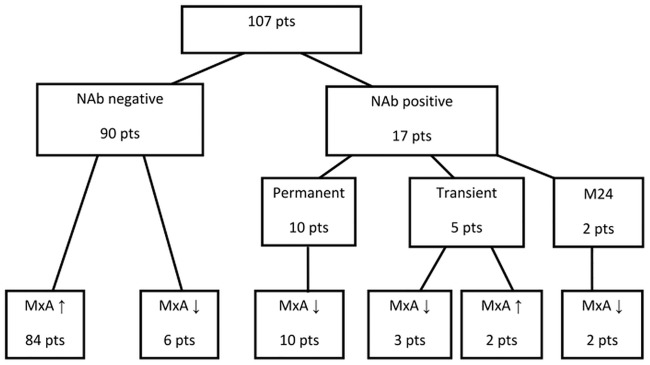
Flowchart of the study. MxA ↑ - mRNA MxA above the cut off (160), MxA ↓ - mRNA MxA below the cut off (160).

NAb positivity was observed in 17 patients (16%), for the majority this was registered for the first time at month 12 or 18 (Fig 1).

**Fig 2:**
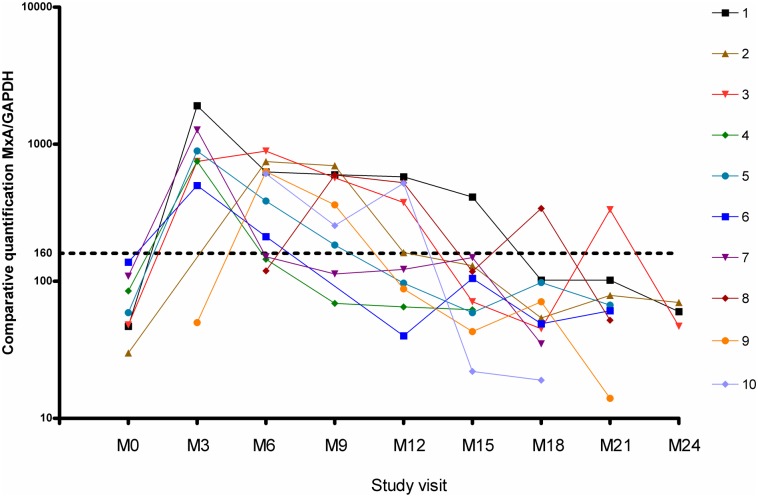
mRNA MxA kinetics in permanent NAb positive patients. MxA/GAPDH = expression of mRNA MxA normalized to the expression level of the housekeeping gene GlycerAldehyde-3-Phosphate Dehydrogenase, GAPDH. M0 = baseline, M3 = month 3, M6 = month 6, etc.

In 10 patients NAb positivity was permanent—five of these patients were treated with IFNβ-1a s.c. (11% of all patients treated with IFNβ-1a s.c.) and five with IFNβ-1b s.c. (13% of all patients treated with IFNβ-1b). The NAb titres in permanently NAb positive patients varied from 33–3000 TRU/ml. Permanent NAb positivity (10 patients) was always accompanied by a drop in MxA mRNA (Fig 2).

In patients transiently positive for NAbs (five subjects, titres 20–163 TRU/ml), MxA mRNA decreased below the cut off in only three cases.

In two patients, positivity for NAbs and a decrease in MxA mRNA was not detected until month 24, thus the division into permanent or transient NAb categories was not possible.

A decrease in MxA mRNA level below the cut off was, on the whole, observed in 19 patients. NAbs were detected in 13 of them (of these, 10 patients were permanently NAb positive and three transiently (Fig 1)).

**Fig 3:**
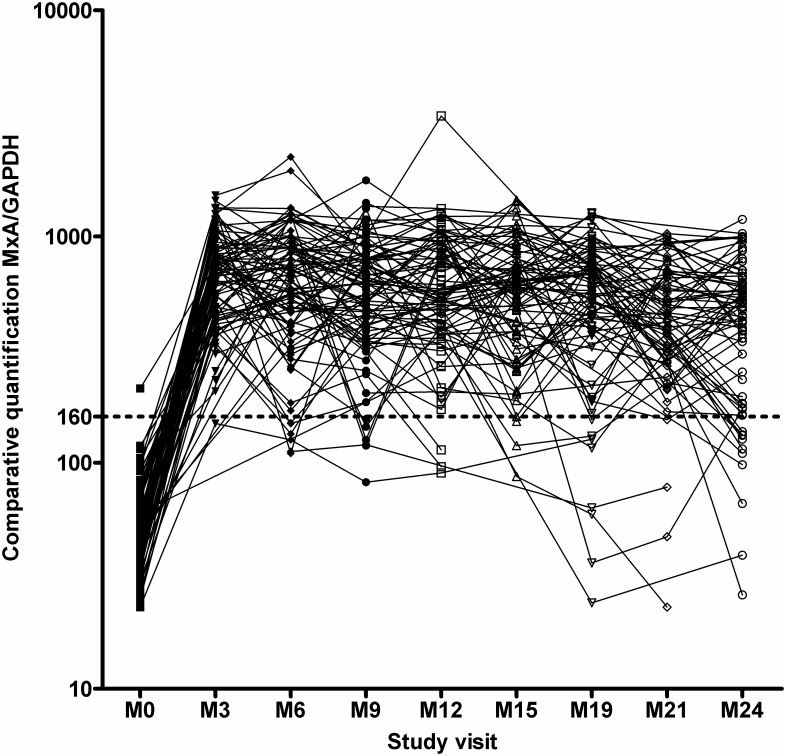
mRNA MxA kinetics in NAb negative patients. MxA/GAPDH = expression of mRNA MxA normalized to the expression level of the housekeeping gene GlycerAldehyde-3-Phosphate Dehydrogenase, GAPDH. M0 = baseline, M3 = month 3, M6 = month 6, etc.

In six patients the decrease in MxA mRNA below the cut off value was not associated with positivity for NAbs (Fig 1). However, the average MxA mRNA value remained above the cut off for the whole period of follow-up in NAb negative patients (Fig 3).

### Correlation between MxA and disease activity

The risk of relapse in patients with a drop in MxA mRNA (n = 19) compared to patients with preserved bioactivity according to their MxA mRNA expression (MxA mRNA above the cut off) was not statistically different.

For analysis of the relationship strictly between MxA mRNA level and clinical activity, only patients with supposed preserved IFNβ bioactivity (n = 90) were included—NAb positive patients (permanent or transient, n = 17) were excluded (Fig 1).

**Fig 4:**
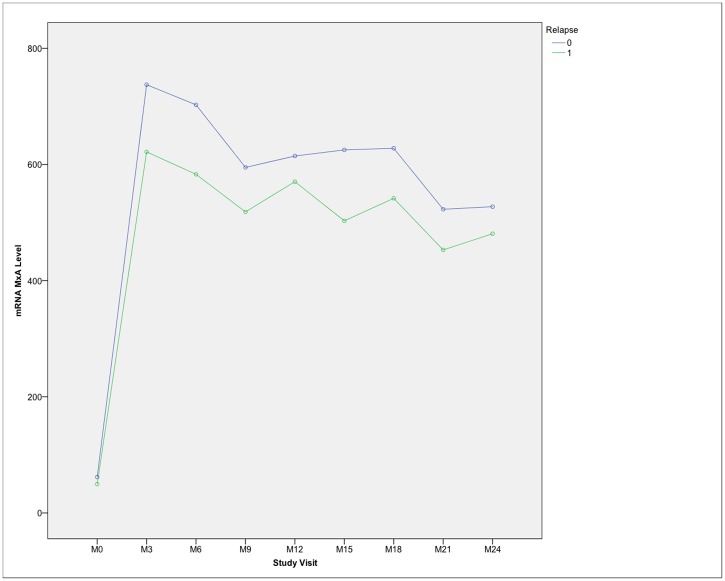
mRNA MxA kinetics in NAb negative patients in relation to clinical activity. M0 = baseline, M3 = month 3, M6 = month 6, etc.

There was a slight trend towards a higher MxA mRNA level in clinically stabilized NAb negative patients, but the mean MxA mRNA level did not significantly differ in patients irrespective of relapse status (Fig 4).

### Baseline MxA—prediction of IFNβ response and clinical outcome

No significant correlation was found between baseline MxA mRNA and month three MxA mRNA levels.

Using survival analysis, none of the various selected baseline MxA mRNA cut off values (see section Statistical analysis) allowed the prediction of time to the first relapse, and there was no correlation between baseline MxA mRNA level and time to the first relapse.

Just as there was no correlation found between MxA mRNA level in month three and time to the first relapse.

There was no significant difference in the baseline MxA mRNA values in patients who subsequently became NAb positive compared to patients who remained NAb negative for the whole period of the study.

## Discussion

In our study, the baseline MxA mRNA value did not predict the response to IFNβ and did not correlate with the clinical activity of the disease during treatment. MxA mRNA as a marker of biological responsiveness to IFNβ treatment proved to identify a substantial number of NAb positive patients (immunopharmacological non-responders). In NAb negative patients, there was no correlation between the MxA mRNA level and clinical disease activity.

MxA and other IFN stimulated genes have a certain level of activity prior to the start of IFNβ treatment, which correlates with endogenous IFN activity. The characteristics of the intrinsic IFN-induced gene expression are called the IFN signature [13]. The specific and characteristic IFN signature has been reported to denote clinical severity in several systemic autoimmune diseases [18; 19].

In treatment naive MS patients, suppression of endogenous IFN-signaling has been reported [20, 21]. In the van der Voort study, MxA mRNA expression was shown to be lower in treatment naive MS patients compared to healthy controls. Moreover, clinically active, treatment naive MS patients had distinctly lower MxA mRNA expression than clinically stable, non-treated MS subjects [20].

In IFNβ treated MS patients, the baseline MxA mRNA (as a part of the IFN signature) has been recently reported to predict further clinical activity, i.e. a lower MxA mRNA level before treatment should correlate with a longer time until the next relapse [13]. However, in our study, MxA mRNA baseline level was not a predictor of the clinical response to IFNβ treatment.

The possibility of baseline MxA mRNA being able to predict clinical disease activity in IFNβ treated MS patients is unlikely for a number of reasons. MS is a heterogeneous disease with individual disease activity. Patients optimally responsive to the therapy could be patients with only a mild clinical course of the disease. Patients with a clinically similar disease course may have different intrinsic modes of immune status [21]. Moreover, in our experience MxA mRNA level fluctuates between investigations and repeated measurements are vital to account for this. Furthermore, repeated measurements should be performed at baseline otherwise the baseline cut off expression cannot be relied upon. As IFNβ plays a crucial role in viral defence [22], the level of MxA mRNA also reflects any ongoing, even subclinical, viral infections [23].

Besides the clinical course, the baseline IFN gene activity status prior to the start of the therapy was reported to influence gene activity in response to exogenous IFNβ treatment. Low expression of MxA mRNA at baseline has been reported to reflect an increased capacity for MxA mRNA induction in response to exogenous IFNβ and possibly a better response to the treatment [10; 13]. In our study, there was no correlation between the treatment naive MxA mRNA values and MxA mRNA values obtained three months after the treatment initiation, thus we cannot support this hypothesis.

Considerably high inter-individual variability in IFNβ responsiveness has been reported in several other studies [24; 25; 26]. It is feasible that analysis of a combination of IFN response genes would be a more reliable predictor than a one-time level of a single isolated IFN response gene [11]. However, even in patients with a high baseline of an IFN response gene set (including high baseline MxA mRNA level), it is not possible to distinguish if their subsequent clinical non-responsiveness is due to lack of response to IFNβ or instead, due to a more active disease initially, or a combination of these factors [27].

### Longitudinal analysis of IFNβ treated patients

The group of biological non-responders (according to our definition) consisted of all the patients with decreased IFNβ bioactivity. In most cases this was caused by NAb positivity (immunopharmacological non-responders). However, not all patients positive for NAbs demonstrated a decrease in MxA mRNA level and vice versa, not all patients with MxA mRNA decrease had NAbs.

In this context, the group of NAb negative patients is a heterogenous group; in the majority of these patients, MxA mRNA stayed above the cut off for the whole follow-up, however, in a few NAb negative subjects, MxA mRNA fell below the cut off (Fig 1).

The decrease in MxA mRNA in NAb negative patients could be caused by several factors. In another study, a third of patients with decreased MxA mRNA presented with IFNβ binding antibodies (BAbs) even though, NAbs could not be identified [28]. BAbs develop in the majority of patients in the first months of IFNβ treatment [29; 30] and in some cases can also cause a loss of IFNβ bioactivity [8;28]. Diminished expression of the IFNβ receptor during prolonged IFNβ treatment or non-compliance could be another explanation for non-antibody mediated abolished biological activity [8; 31; 32].

Recent data have confirmed the relationship between IFNβ bioactivity (MxA mRNA level) during treatment and clinical disease activity [5]. Without biological activity, the clinical effect of IFNβ is unlikely and a drop in MxA mRNA below the cut-off level unequivocally increases the risk of relapse. However, even though an MxA mRNA level above the cut off identifies the patient as an IFNβ responder, the actual level of disease activity does not correlate with the MxA mRNA level. No significant difference in MxA level between clinically active and clinically stabilized NAb negative patients was revealed. Thus, in patients with preserved biological activity, MxA mRNA level cannot identify who is at higher risk of disease progression.

There are a number of limitations in our study. In patients showing decreased MxA mRNA, but neither NAbs nor BAbs, the MxA mRNA induction test should be used to confirm the responsiveness of patients to IFNβ [4; 28]. This allows discrimination of patient non-compliance and was not consistently performed in all patients when MxA mRNA dropped below the cut off level. The relatively short follow up is another limitation. Two patients in our study did not become NAb positive (with a drop in MxA mRNA level below cut off) until month 24, the end of our study. Thus, MxA mRNA kinetics were not fully assessed in these patients.

Fluctuations in MxA mRNA levels only became clear during the follow-up, thus we did not perform repeated MxA mRNA measurements for assessing the baseline MxA mRNA cut off.

### Conclusion

In conclusion, in IFNβ treated patients, MxA mRNA represents a primary tool for monitoring the IFNβ biological effect in everyday clinical practice. However, our study shows that the risk of disease activity in biological responders is not unequivocally predictable using MxA mRNA level. Furthermore, the relevance of baseline MxA mRNA values in predicting IFNβ response is ambiguous and should be further elucidated.
